# [Retracted] Hypoxic preconditioned bone mesenchymal stem cells ameliorate spinal cord injury in rats via improved survival and migration

**DOI:** 10.3892/ijmm.2025.5672

**Published:** 2025-10-22

**Authors:** Weiheng Wang, Xiaodong Huang, Wenbo Lin, Yuanyuan Qiu, Yunfei He, Jiangming Yu, Yanhai Xi, Xiaojian Ye

Int J Mol Med 42: 2538-2550, 2018; DOI: 10.3892/ijmm.2018.3810

Following the publication of the above paper, it was drawn to the Editor's attention by a concerned reader that certain of the data panels (namely, three of the six panels) in Fig. 3 showing the results of migration assay experiments were strikingly similar to data in a paper which was submitted for publication at around the same time by the same research group to the journal *Stem Cells International*, where the results were described differently. Upon performing an independent analysis of the data in this paper in the Editorial Office, it came to light that data included in Figs. 1C, 6D and 7B-D were also strikingly similar to data appearing in a few other articles written by the same research group, one of which had already been published and one of which was submitted for publication at around the same time as the above paper. Moreover, two pairs of data panels in Fig. 3 also contained overlapping sections of data, such that data which were intended to show the results of differently performed experiments had apparently been derived from a smaller number of original sources.

Given the apparent re-use of a large number of the data featured in the above paper in other articles by the same research group, and in view of the overlapping data identified in Fig. 3, the Editor of *International Journal of Molecular Medicine* has decided that this paper should be retracted from the Journal on account of a lack of confidence in the presented data. The authors were asked for an explanation to account for these concerns, but the Editorial Office did not receive a reply. The Editor apologizes to the readership for any inconvenience caused.

